# A Polymorphism Affecting MYB Binding within the Promoter of the *PDCD4* Gene is Associated with Severe Asthma in Children

**DOI:** 10.1002/humu.22340

**Published:** 2013-05-20

**Authors:** Aristea Binia, Nicole Van Stiphout, Liming Liang, Sven Michel, Pankaj K Bhavsar, K Fan Chung, Chris E Brightling, Peter J Barnes, Michael Kabesch, Andrew Bush, William OC Cookson, Miriam F Moffatt

**Affiliations:** 1 Molecular Genetics and Genomics Section, National Heart and Lung Institute, Imperial College LondonLondon, United Kingdom; 2 Nestlé Research Centre, Nutrition and Health DepartmentVers-chez-les-Blanc, Lausanne, Switzerland; 3 Department of Paediatric Respiratory Medicine, National Heart and Lung Institute, Imperial College LondonLondon, United Kingdom; 4 Department of Epidemiology, Department of Biostatistics, Harvard School of Public HealthBoston, Massachusetts; 5 Department of Paediatric Pneumology and Allergy, University Children’s Hospital Regensburg (KUNO)Germany; 6 Airway Disease Section, National Heart and Lung Institute, Imperial College LondonLondon, United Kingdom; 7 Institute for Lung Health, Department of Infection, Immunity and Inflammation, University Hospitals of LeicesterLeicester, United Kingdom

**Keywords:** *PDCD4*, asthma, severe, childhood, *MYB*

## Abstract

A previous genome-wide association study in asthma revealed putative associations that merit further investigation. In this study, the genome-wide significant associations of SNPs at the 5% false discovery rate were examined in independent groups of severe asthmatics. The panel consisted of 397 severe asthmatic adults, 116 severe asthmatic children, and a collection of 207 family-trios with an asthmatic proband. Three SNPs in the *PDCD4* gene (*rs6585018:G>A*, *rs1322997:C>A*, and *rs34104444:G>A*) were significantly associated with severe childhood asthma (*P* values: 0.003, 0.002, 0.004) and total immunoglobulin E (IgE) levels (*P* values: 0.034, 0.041, 0.052). In an independent group of 234 asthmatic children and 652 controls, *PDCD4* SNPs *rs1407696:T>G* and *rs11195360:T>C* were associated with total IgE levels (*P* values: 0.006, 0.014). *In silico* analysis of *PDCD4* locus showed that *rs6585018:G>A* had the potential to affect MYB transcription factor binding, shown to act as a PDCD4-transcription inducer. Electromobility shift assays and reporter assays revealed that *rs6585018:G>A* alters MYB binding thereby influencing the expression of PDCD4. SNPs within *MYB* itself confer susceptibility to eosinophilia and asthma. Our association between a variant MYB binding site in *PDCD4* and the severest form of childhood asthma therefore suggests that PDCD4 is a novel molecule of importance to asthmatic inflammatory responses.

## Introduction

Asthma is a chronic inflammatory disease of the lungs involving a number of physiological mechanisms. It affects approximately 300 million people worldwide and is the single most common respiratory disease of childhood [Masoli et al., 2004]. Because of the frequent hospital admissions and the regular use of antiasthma treatments, approximately 80% of the entire financial burden for the disease is attributable to the 20% of patients with the severest, steroid resistant form of the disease [Smith et al., 1997].

During the last decade, genome-wide association studies (GWAS) on asthma phenotypes have highlighted novel putative pathways adding novel targets to the list of asthma-associated loci [Zhang et al., 2012]. It is estimated, however, that the largest part of the heritability of complex diseases remains unidentified [Manolio et al., 2009]. Some of the proposed explanations for the “missing heritability” include the small effect size of numerous variants not reaching a genome-wide significant association at the large GWAS, the existence of rare variants not present in commercial genotyping arrays and the heterogeneity of the investigated disease phenotypes [Gibson, 2010; Manolio et al., 2009; Yang et al., 2010]. In addition, there are only a small number of studies providing a functional evaluation of the GWAS findings [Lluis et al., 2011; Verlaan et al., 2009]. Studies combining genetic analysis in homogenous populations and incorporating functional data could also evaluate or propose novel unexplored candidate loci assessing simultaneously a well-defined phenotype of the disease [Cusanovich et al., 2012; Tantisira et al., 2011].

Previously, a GWAS for asthma identified a locus on chromosome 17q21, containing the *ORMDL3* (MIM #610075) and *GSDMA* genes (MIM #611218), to be highly significantly associated with childhood asthma [Moffatt et al., 2007]. This association has now been widely replicated by a number of independent studies [Binia et al., 2011; Bouzigon et al., 2008; Galanter et al., 2008; Madore et al., 2008; Moffatt et al., 2010; Tavendale et al., 2008]. On-going functional studies aim to elucidate the biological role of these findings [Breslow et al., 2010; Cantero-Recasens et al., 2010].

**Figure 1:**
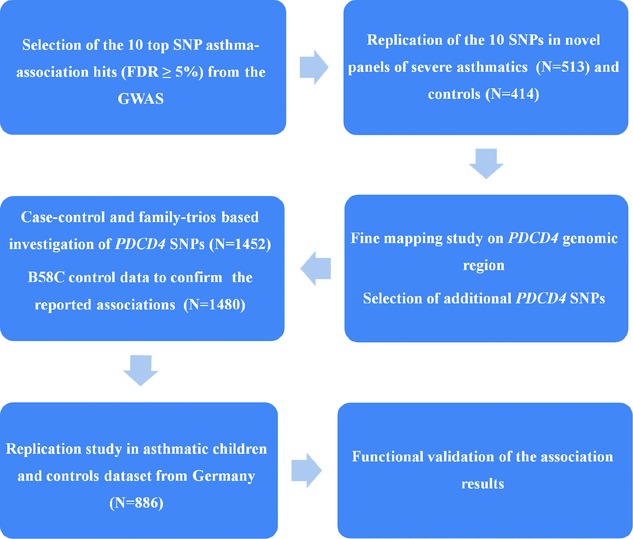
The outline of the study plan (N: number; FDR: false discovery rate; B58C: British 1958 Birth Cohort study).

In addition to the 17q21 locus exceeding the genome-wide significance level (at 1% false discovery rate, FDR), genetic markers showed suggestive results at 5% FDR [Moffatt et al., 2007]. Evidently a great proportion of these represent false positive results [McCarthy et al., 2008]; however some of these hits could point to further asthma-associated loci with a smaller effect not captured by the GWAS. This study aimed to further investigate these underlying associations in cases of child and adult severe asthma followed by fine-mapping and functional assays (Fig. 1).

## Material and Methods

### Subjects, Genotyping, and Imputation

#### Subjects from the United Kingdom

Cases, adults, and children all white with British ancestry were recruited from severe asthma clinics based within the UK. For the severe asthmatic adults, asthma was physician-diagnosed and defined as severe according to the American Thoracic Society (ATS) criteria (2000). For the child cases, the Global Initiative for Asthma (GINA) criteria were followed [Bousquet, 2000] with severe asthma defined as Step 4 severe/persistent asthma which includes patients with continuous symptoms during the day, frequent during the night and Forced Expiratory Volume in 1 sec (FEV_1_)</ = 60%. Mild asthmatic group included young adults and children (Age: mean [standard deviation] = 29.49 [8.10]), corticosteroid-naive, receiving treatment with only inhaled β_2_-agonists in an intermittent basis. Additionally, a panel of 207 families administered a standard questionnaire (based on the ATS and International Study of Asthma and Allergies in Childhood, ISAAC questionnaires) and recruited through a proband with severe asthma (Step III asthma or worse) according to the British Thoracic Society guidelines were included in the study [Moffatt et al., 2007]. Phenotypic characterization of the cases and controls included detailed clinical data, lung function tests, bronchial hyperresponsiveness, total IgE and blood eosinophils counts. Three hundred and ninety seven severe asthmatic adults, 111 mild adult asthmatics and 116 severe asthmatic children were genotyped for the selected SNPs. DNA was extracted from whole blood samples using the Wizard® Genomic DNA Purification Kit (Promega; http://www.promega.com) and from saliva using the Oragene®·DNA collection system (DNA Genotek, http://www.dnagenotek.com). DNA samples were quantified using NanoDrop® ND-1000 UV-Vis Spectrophotometer. TaqMan® SNP Genotyping Assays (Applied Biosystems; http://www.appliedbiosystems.com, 7300 Real-Time PCR System) were used for genotyping (assay details available upon request). Only for SNP *rs1322997:C>A*, genotyping data from 1,480 asthma-free healthy controls from the British 1958 Birth Cohort study (http://www.b58cgene.sgul.ac.uk) were available and they were used as controls compared with severe asthmatic children to confirm the genetic associations. No descriptive data were available from the British 1958 Birth Cohort study.

#### Subjects from Germany

The dataset consisting of two distinct cohorts has been previously described [Moffatt et al., 2007]. Asthma cases were recruited from the Multicentre Asthma Genetics In Childhood Study (MAGICS), whereas subjects from the International Study of Asthma and Allergy in Childhood, phase II (ISAAC II) [Weiland et al., 1999] served as controls. Details of the recruitment, chip-genotyping (Illumina HumanHap300) and definition of phenotypes have been previously described [Michel et al., 2010; Toncheva et al., 2012]. To select the severe asthmatics from the initial cohort we restricted the analysis to those asthmatics that reported at least one (Severe Asthma 1, *N* = 234) or at least four (Severe Asthma 2, *N* = 104) hospital visit due to asthma within the last 12 months before recruitment. Control subjects (*N* = 652) were negative for asthma. Total serum IgE levels were measured and the log-transformed values were used for the association analysis.

Study genotypes were imputed using the current two stage approach, separating phasing of study data and the subsequent imputation [Howie et al., 2012]. First prephasing of the study genotypes was done with MaCH [Li et al., 2010]. Second minimac [Howie et al., 2012] was used with the recommended settings [http://genome.sph.umich.edu/wiki/Minimac:_GIANT_1000_Genomes_Imputation_Cookbook] utilizing the 1000G Phase I Integrated Release Version 3 Haplotypes [http://www.sph.umich.edu/csg/abecasis/MaCH/download/1000G.2012–03–14.html] as refere-nce panel.

### Statistical Analysis

Deviation from Hardy–Weinberg equilibrium was calculated for the allele frequencies for both cases and controls by a *χ*^2^ test. SNPs with allele frequencies presenting a significant deviation from Hardy–Weinberg equilibrium were excluded. For the SNPs not previously part of the GWAS SNP chip [Moffatt et al., 2007], a family-based test (the Transmission Disequilibrium Test, TDT) was performed for the family panel in R statistical package. Allele frequencies were compared between severe asthmatics (cases) and nonasthmatic subjects (controls) by Fisher’s exact test and odds ratios (OR) calculated for minor alleles. The same comparisons were performed for severe asthmatics (cases) versus mild asthmatics (controls). At each stage of the analysis, the 5% FDR adjustments for multiple testing were calculated at Qvalue software in R statistical package [Storey and Tibshirani, 2003]. For quantitative traits (total IgE levels, blood eosinophils counts and FEV_1_%) and SNP associations, an analysis of variance was performed using log-transformed variables to achieve a normal distribution. Haplotype analysis was carried out for the cases and the controls in the severe asthmatic children versus nonasthmatic children study design using Haploview 3.3 [Barrett et al., 2005]. Plots were generated in R statistical package. For the replication panel from Germany, additive genetic effects were modeled using logistic or linear (serum IgE levels) regression implemented in the ProbABEL software package [http://www.genabel.org; Aulchenko et al., 2010].

### Fine Mapping of the PDCD4 Region

**Figure 2:**
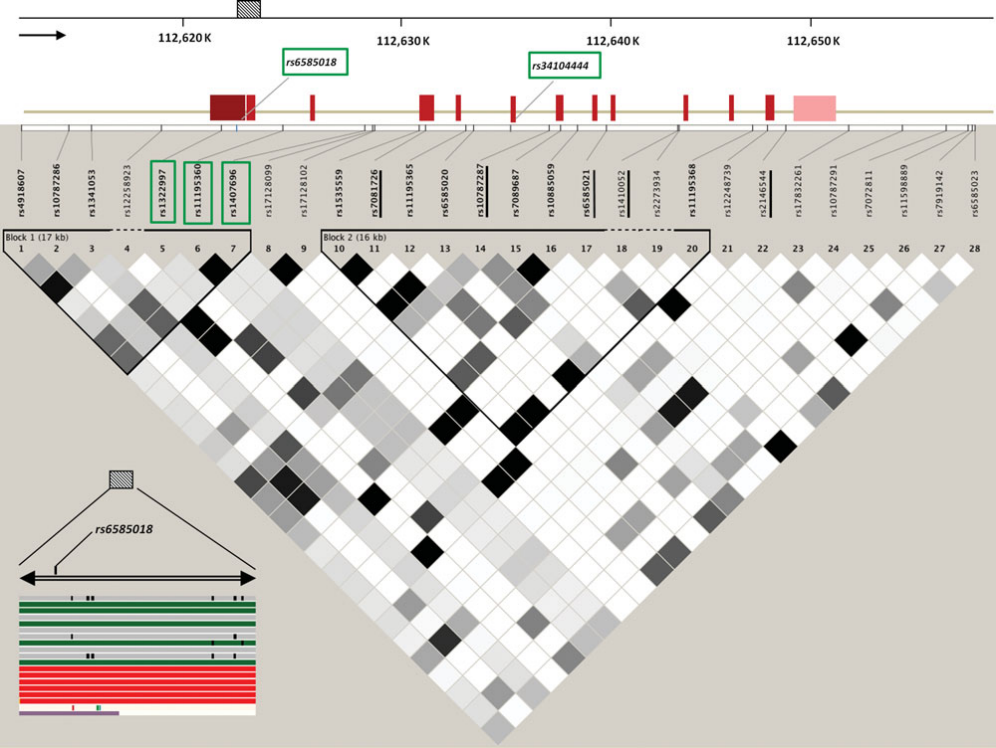
The *PDCD4* gene structure. Haplotype analysis results from the HapMap CEU genotype data (combined Phase I and II) are shown in a color scale map plot expressing the *r*^2^ value for linkage disequilibrium (white: *r*^2^ = 0, black: *r*^2^ = 1). Underlined SNPs were included in the original GWAS study [Moffatt et al., 2007]. SNPs selected in the present study are shown in a frame box. The promoter area 112,621,625 to 112,622,604; NCBI 36.1 (chr10:112,631,200 to 112,632,179; NCBI 37.3) sequenced in the fine mapping study is indicated by the patterned box. The area was entered in the Ensembl Genome Browser (http://www.ensembl.org/index.html) to identify putative regulatory elements in different cell lines (red/green lines: predicted promoter transcription; purple lines: polymerase III-associated region; grey lines and black boxes: unidentified regulatory elements).

The area including the SNPs on *PDCD4* (NM_145341.3) genotyped in the original GWAS [Moffatt et al., 2007] was examined and tagging SNPs covering variations not included in the arrays used in the original GWAS were selected using the pair-wise tagging algorithm in Haploview 3.3 (*r*^2^ > 0.8) [Barrett et al., 2005]. Linkage disequilibrium (LD) of the area was assessed using the HapMap CEU genotype data (version 2, Phase 1 and 2, http://hapmap.ncbi.nlm.nih.gov/). Genome browsers http://www.ensembl.org/index.html and http://genome.ucsc.edu/ were also used to visualize the LD and the regulatory elements as reported from the Encyclopedia of DNA Elements (ENCODE) project spanning the gene region [Kuhn et al., 2012]. Three additional tagging SNPs and one coding SNP in the *PDCD4* (MIM #608610) area (*rs1322997:C>A*, *rs11195360:T>C*, *rs1407696:T>G*, and *rs34104444:G>A*) were selected to be genotyped in the fine mapping study (Fig. 2). In addition, the putative promoter of *PDCD4* was sequenced for the identification of potentially novel polymorphisms in 24 samples with known *rs6585018:G>A* genotypes (14 AA and 10 GA). Two sets of primers were designed (Invitrogen; http://www.invitrogen.com, sequences available upon request) to amplify 2 promoter regions, 112,621,625 to 112,622,006 and 112,622,164 to 112,622,604 (NCBI Build 36.1). The sequencing results were assembled aligned and visualized using the CodonCode Aligner software Version 2.06 (http://www.codoncode.com/).

#### Cell culture, protein extraction, and electromobility shift assays

To obtain protein extracts for the electromobility shift assays (EMSA) experiment, Jurkat (T cells), Daudi (B cells) and A549 (Airway epithelial cells) were purchased from the American Type Culture Collection (http://www.atcc.org). Jurkat, A549 and Daudi cell lines were cultured in standard media at 37°C and 5% CO_2_. Cell lines were not allowed to exceed passage 5 or 6 before protein extractions were prepared using a modified Schreiber protocol [Schreiber et al., 1989]. Yield of protein obtained was quantified using the Bradford assay [Bradford, 1976]. The regions containing the *PDCD4* polymorphisms were analyzed for transcription factor binding sites using TFSEARCH [Heinemeyer et al., 1998] and MatInspector [Cartharius et al., 2005]. The expression of the protein in Jurkat, in A549 and Daudi cell lines was confirmed by Western blots (data not shown). For EMSA on *rs6585018:G>A*, sense and antisense single-stranded oligonucleotides for the two alleles were designed (oligonucleotides available upon request) and synthesized (Invitrogen; http://www.invitrogen.com). MYB consensus binding sequence (5′-YAAC[GT]G-3′) oligonucleotides were used as a positive control. The oligonucleotides were annealed to form the SNP-specific probes and labeled by Klenow fragment (Invitrogen) with α-^32^P CTP (PerkinElmer®; http://www.perkinelmer.com) [Hacking et al., 2004]. EMSA binding reactions were set up containing 4–10 μg of nuclear extracts, 2–3 μl of radiolabeled probe, 12 mM HEPES buffer (pH 7.8), 100 mM NaCl, 1 mM EDTA, 1 mM EGTA, 12% glycerol, and 0.5 μg poly(dA-dT) (Sigma; http://www.sigmaaldrich.com) in a final reaction volume of 15 μl. Competition assays were included adding unlabeled probe 15 times (15×) and 60 times (60×) of the amount of the labeled probe. For supershift assays, 0.3 ng of antibody was added before incubation. The antibodies used were anti-MYB and anti-SRY (Abcam, http://www.abcam.com). After incubation, the reactions were run on 6% nondenaturing polyacrylamide gels at 4°C and 80–100 V, using 0.5× TBE running buffer. Kodak X-Omat AR film autoradiography (Sigma) was used to visualize the results.

#### Constructs, transfections, reporter assays

pGL3.*rs6585018*+A and pGL3.*rs6585018*+G constructs were generated by amplifying a 219 bp sequence spanning *rs6585018:G>A* (sequences available upon request) and cloning into a *MluI-BglII*-digested pGL3-promoter vector (Promega) upstream of the SV40 promoter. The constructs were verified by multiple digestions and sequencing (data not shown). Nucleofector (Lonza; http://www.lonza.com) and electroporation (Nucleofector® II Device) were used to transfect Jurkat cell lines with and without the reporter gene constructs (triplicates done for each experiment) following the optimized manufacturer’s protocol for Jurkat cell lines (ATCC). One million cells were transfected with 2 μg of total DNA. Vector pRL.TK was cotransfected to normalize for transfection efficiency. After 24 hr, cells were lysed and analyzed using the Dual-Luciferase Reporter Assay kit (Promega) and a luminometer following the manufacturer’s protocol.

## Results

### Top Ten GWAS Hits Association Study

From the previous GWAS [Moffatt et al., 2007], the top ten hits (at FDR) ≥ 5%) were selected, excluding the 17q21 region, for genotyping in severe asthmatics (*N* = 513) and healthy controls (*N* = 414) (Fig. 1, Table 1). Call rates and tests for deviation from Hardy–Weinberg Equilibrium results for all SNPs showed that 9 out of 10 SNPs passed the genotyping quality control (Table 2). SNP *rs1401107*:C>T had a call rate <90% and was therefore excluded from further analyses.

**Table 1:** Characteristics of the UK Sample Collections Included in the Study

	Severe asthmatics		Family trios (*N* = 207) dataset					
	Severe	Severe						B58C
	asthmatic	asthmatic	Asthmatic	Asthmatic	Healthy	Healthy	Mild	Healthy
	adults	children	adults	children	adults	children	asthmatics	controls
Number	*N* = 397	*N* = 116	*N* = 119	*N* = 295	*N* = 269	*N* = 145	*N* = 111	*N* = 1480
Mean age (yr) (SD)	47.17 (13.85)	12.05 (2.80)	35 (10.26)	10 (3.15)	40.92 (8.09)	10.11 (3.58)	27.85 (5.74)	–
Sex (F:M)	2:1	2:3	1:1	2:3	1:1	1:1	1:2	
IgE (kU/l) (SD)	258.6 (464.9)	1204 (2829.6)	264.44 (465.8)	725.01 (1068)	123.14 (343.50)	241.39 (517.02)	–	
Eosinophils counts (10^9^/l) (SD)	–	0.64 (0.63)	0.32 (0.22)	0.69 (0.45)	0.22 (0.21)	0.40 (0.36)	–	
FEV_1_%	64.3 (19.6)	76.8 (18.49)	–	–	–	–	90.0 (13.2)	

SD, standard deviation; F/M, female/male; IgE, immunoglobulin E; yr, years; l, Litre; FEV_1_%, forced expiratory volume in 1 sec; B58C**,** British 1958 Birth Cohort study.

**Table 2:** Genotype Frequencies and *P* Values for *rs6585018:G>A* in Severe Asthmatic Children and Adults Groups

						Case–control analysis			
						MAF		Minor versus major allele	
SNP Ancestral>			GWAS-	Call success	HWE χ^2^	Cases	Controls		
Mutant	Chrom	Gene	log_10_(*P*)	rates	*P* value	(*N* = 513)	(*N* = 414)	OR (CI)	*P* value
*rs1401107:C>T*	2	Intergenic	5.92	85%	0.07	NA			
*rs10270097:C>A*	7	*DGKI*	5.24	96.3%	0.38	0.07	0.06	1.32	0.08
								(0.88–1.98)	
*rs481297:T>C*	18	*ST8SIA5*	5.09	95.2%	0.10	0.34	0.34	1.00	0.49
								(0.78–1.28)	
*rs2243603:G>C*	20	*SIRPB1*	4.33	95.9%	0.37	0.23	0.25	0.88	0.18
								(0.67–1.16)	
*rs1356847:T>C*	2	Intergenic	4.15	94.8%	0.98	0.43	0.31	1.06	0.32
								(0.83–1.39)	
*rs12715305:C>G*	3	*DLEC1*	4.03	96.3%	0.99	0.32	0.33	0.94	0.32
								(0.74–1.21)	
*rs11097415:G>A*	4	*SHROOM3*	4.01	98.3%	0.95	0.38	0.38	1.00	0.49
								(0.78–1.26)	
*rs6656822:T>C*	1	*SLC19A2*	4.00	91.5%	0.93	0.30	0.31	0.94	0.32
								(0.72–1.22)	
*rs6585018:G>A*	10	*PDCD4*	4.70	99.6%	0.95	0.05	0.03	1.92	0.006
								(1.14–3.32)	
*rs248944:C>G*	19	*ZNF506*	4.23	97.1%	0.97	0.36	0.38	0.91	0.17
								(0.75–1.11)	

Chrom, chromosome; HWE, Hardy–Weinberg Equilibrium; N, absolute number; MAF, minor allele frequency; NA, not analyzed; OR, odds ratio; CI, confidence intervals; *DGKI,* diacylglycerol kinase iota; *ST8SIA5,* ST8 alpha-N-acetyl-neuraminide alpha-2,8-sialyltransferase 5; *SIRPB1,* signal-regulatory protein beta 1; *DLEC1*, deleted in lung and esophageal cancer 1; *SHROOM3,* shroom family member 3; *SLC19A2,* solute carrier family 19 (thiamine transporter), member 2; *PDCD4,* programmed death cell domain 4; *ZNF506,* zinc finger protein 506.

Analyses of severe asthmatics versus nonasthmatic controls revealed that 1 of the 9 loci from the initial GWAS showed evidence of association in this independent group of cases and controls. We discovered a significant association between the marker *rs6585018:G>A* and severe asthma (OR = 1.92, 95% Confidence Interval, [95% CI]: 1.14–3.32 and *P* = 0.006) (Table 2). *Rs6585018:G>A* is located within the predicted promoter of the *Programmed Death Cell Domain 4* (*PDCD4*) gene. Taking into account the heterogeneity of the phenotypes, childhood asthma cases and controls were separated from adulthood asthma cases and controls to test the association for *rs6585018:G>A*. The results showed that the association was restricted to the childhood asthma group (OR = 2.91, 95% CI: 1.40–6.05, *P* = 0.003). Risk allele G was more frequent in the severe asthmatic children group (0.11) compared with controls (0.04) and also compared with the general European population (CEU: HapMap, *rs6585018:G>A*, minor allele frequency [MAF]: 0.06; http://www.ncbi.nlm.nih.gov/ and EUR: 1000 Genomes Project, *rs6585018:G>A*, MAF: 0.05; http://www.1000genomes.org/) (Table 3). No significant associations were seen for severe asthmatic adult patients (OR = 0.94, CI: 0.53–1.69, *P* = 0.48).

**Table 3:** Association Test Results for all Population Groups for *PDCD4* SNPs

	Severe asthmatic children:		Severe asthmatic children:		Family trios,					Severe asthmatic children:	
SNP:	Healthy children		Mild asthmatic children		TDT		Association with		Association with	Controls including	
Ancestral>Mutant	(116:145) (116 vs. 145)		(116:111) (116 vs. 111)		(*N* = 207 families)		*PDCD4* mRNA levels^a^		IgE levels^b^	B58C data (116:1,624)	
	Risk allele	OR (CI), *P* value	Risk allele	OR (CI), *P v*alue	*χ*^2^	*P* value	LOD	*P* value	*P* value	Risk allele	OR (CI), *P* value
*rs1322997:C>A*	0.11:0.04	2.94 (1.41–6.11), *P* = 0.002	0.11:0.03	4.34 (1.76–10.57), *P* = 0.001	14	*P* = 0.0002	2.64	*P* = 0.008	*P* = 0.041	0.11:0.04	2.73 (1.73–4.13), *P* = 0.0001
*rs6585018:G>A*	0.11:0.04	2.91 (1.40–6.05), *P* = 0.003	0.11:0.03	3.77 (1.57–8.88), *P* = 0.002	14	*P* = 0.0002	2.43	*P* = 0.008	*P* = 0.034	NA	
*rs11195360:T>C*	0.35:0.29	1.31 (0.87–1.97), *P* = 0.11	0.35:0.35	0.98 (0.66–1.46), *P* = 0.48	0.5	*P* = 0.47	0.01	*P* = 0.809	*P* = 0.077	0.35:0.30	1.24 (0.91–1.69), *P* = 0.25
*rs1407696:T>G*	0.36:0.28	1.46 (0.95–2.11), *P* = 0.08	0.36:0.35	1.05 (0.51–1.21), *P* = 0.49	1.0	*P* = 0.30	0.002	*P* = 0.465	*P* = 0.051	0.36:0.29	1.36 (0.99–1.84), *P* = 0.07
*rs34104444:G>A*	0.10:0.04	2.83 (1.31–6.11), *P* = 0.004	0.10:0.03	3.52 (1.45–8.38), *P* = 0.003	11	*P* = 0.0009	2.59	*P* = 0.007	*P* = 0.052	NA	

*P* values were calculated for allele frequencies using Fisher’s exact test. Severe asthmatics were at Step IV severe/persistent asthma and family trio asthmatic probands were at Step III or more asthma. Corrected *P* values for multiple testing are presented.

CI, confidence intervals; LOD, logarithm of odds ratio; TDT, transmission disequilibrium test; NA, not analyzed; B58C, British 1958 Birth Cohort Study.

Association results between the SNPs and the transcript levels can be found at http://www.sph.umich.edu/csg/liang/asthma.

IgE values were log-transformed.

### Fine Mapping on PDCD4 Region

To fine-map the genetic association in detail, additional SNPs were selected for genotyping (Fig. 2). First, resequencing of the putative *PDCD4* promoter in 24 individuals (of known *rs6585018:G>A* genotypes) identified no additional novel SNPs (sequence reads available upon request). In the next step, LD analysis was conducted to identify tagging SNPs within the *PDCD4* gene capturing polymorphisms not genotyped nor tagged in the original GWAS, so that all polymorphisms spanning the entire *PDCD4* area are adequately captured by GWAS-genotyped SNPs and those included in the present study (Fig. 2). The four SNPs selected (Supp. Table S1) were genotyped in the severe asthmatic children (*N* = 116). All *PDCD4* SNPs, including *rs6585018:G>A*, were further genotyped in the subjects from the family collection panel that included both asthmatics (*N* = 414) and healthy controls (*N* = 414), and mild asthmatic young adults and children (*N* = 111) (Table 1). All SNP genotype frequencies were in Hardy-Weinberg equilibrium as assessed by *χ*^2^ test (Supp. Table S1).

Comparing severe asthmatic children versus healthy controls, in addition to *rs6585018:G>A*, SNPs *rs34104444:G>A*, and *rs1322997:C>A* were significantly associated with severe childhood asthma (OR = 2.83 95% CI: 1.31–6.11, *P* = 0.004 and OR = 2.94 95% CI: 1.41–6.11, *P* = 0.002, respectively) (Table 3). When mild asthmatic young adults were used as controls, stronger associations were seen for all SNPs, with *P* values ranging from 0.001 to 0.003 (Table 3). Using data for 1480 asthma-free healthy controls from the British 1958 Birth Cohort study (http://www.b58cgene.sgul.ac.uk) strong associations (*P* = 0.0001 for *rs1322997:C>A*) were seen for severe asthmatic children (Table 3). The results remained significant when correction for multiple testing was conducted using a FDR threshold of 5%.

The family dataset with a severe asthmatic proband, used in the original GWAS (11) in which an asthma-association with *rs6585018:G>A* was reported (TDT: *P* = 0.0002), also showed significant associations for asthma and SNPs *rs1322997:C>A* and *rs34104444:G>A* (TDT: *P* = 0.0002 and *P* = 0.0009, respectively) (Table 3). As seen in the severe asthmatic children group, the MAFs of all *PDCD4* SNPs were increased in the cases compared with control groups (MAF ∼ 0.07 for the asthmatic probands compared with ∼0.04 for the control group).

Total immunoglobulin E levels were higher for the children carrying the asthma-associated variant G in *rs6585018:G>A* and *rs1322997:C>A, P* = 0.034 and *P* = 0.041, respectively (Table 3 and Supp. Fig. S1). Eosinophils counts and lung function measurements (% Forced Expiratory Volume in 1 sec, FEV_1_%) did not differ between subjects carrying the alternate alleles for any of the examined SNPs (data not shown).

The effect size of the association of *rs6585018:G>A* with asthma was estimated at OR = 1.99 95% CI: 1.07–3.81, *P* = 0.02, when combining all asthmatic children, severe and asthmatic probands from the family dataset (*N* = 411) and comparing them with healthy children (*N* = 145) (Supp. Table S2).

Haplotype analysis for the severe asthmatic and nonasthmatic children showed that the region was in high LD with two LD blocks present (Supp. Fig. S2). Significantly associated with asthma SNPs *rs6585018:G>A*, *rs34104444:G>A*, and *rs1322997:C>A* are part of the same LD block (Supp. Fig. S2).

### Replication Study Results

*PDCD4* SNPs *rs1407696:T>G* and *rs11195360:T>C* were shown to be significantly associated with IgE levels (*P* < 0.01 and *P* < 0.02, respectively) in an independent group of asthmatic children and controls from Germany (Supp. Table S3). No significant associations were observed for any tested SNP with the asthmatic status. SNPs *rs1407696:T>G* and *rs11195360:T>C* belong to a different LD block compared with the SNPs demonstrating significant associations in the UK population (Supp. Fig. S2).

### Functional Analysis of PDCD4 SNPs

Interrogation of transcriptome data previously generated from lymphoblastoid cell lines [Dixon et al., 2007] (http://www.sph.umich.edu/csg/liang/asthma) revealed that all three *PDCD4* SNPs *rs1322997:C>A*, *rs6585018:G>A*, and *rs34104444:G>A* that had shown associations with severe asthma were correlated with the expression levels of *PDCD4* (LOD scores 2.64, 2.43, and 2.59, respectively) (Table 3). The association results between SNPs and *PDCD4* transcript levels are corrected for multiple testing adjusted at a FDR threshold of 5%.

**Figure 3:**
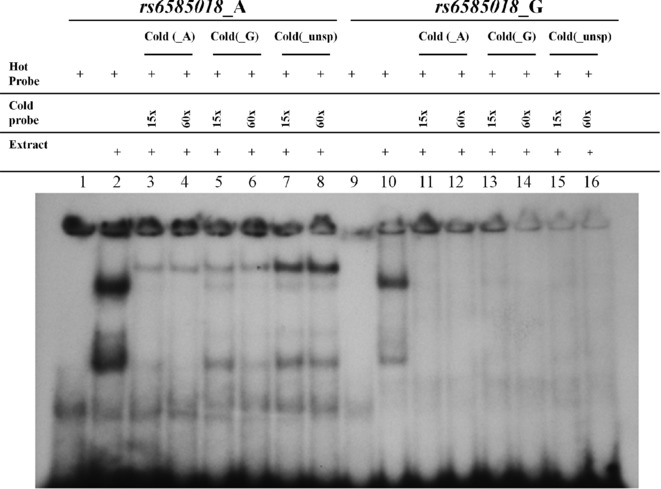
Competition assay for *PDCD4* SNP *rs6585018:G>A* using 10 μg of Jurkat nuclear extract per reaction. Lanes 1 and 9 contain only the hot probes *rs6585018*_A and rs6585018_G, respectively. Lanes 2–8 include the *rs6585018*_A hot probe and lanes 11–16 include the *rs6585018*_G hot probe. Lanes 2 and 10 are reactions with the hot probe and the extract only. Unlabeled probe in excess was added in the competition assays (lanes 3–8 and 11–16) as follows: lanes 3 and 11—15× and lanes 4 and 12—60× of cold *rs6585018*_A, lanes 5 and 13—15× and lanes 6 and 14—60× of cold *rs6585018*_G and lanes 7 and 15—15× and lanes 8 and 16—60× of cold *PDCD4*_unsp (unspecific) probe.

**Figure 4:**
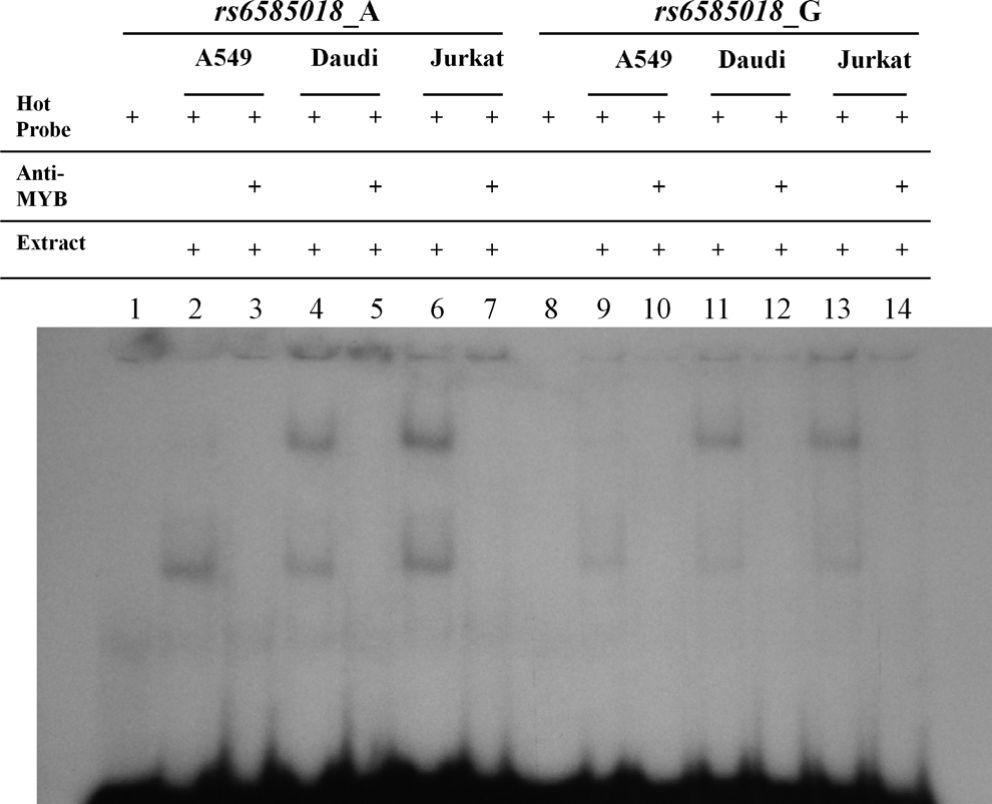
Supershift assays for *PDCD4* SNP *rs6585018:G>A* using 10 μg of either A549, Daudi or Jurkat nuclear extracts. Lanes 1 and 8 contain only the hot probes *rs6585018*_A and *rs6585018*_G, respectively. Lanes 2–7 include the *rs6585018*_A hot probe and lanes 9–14 the *rs6585018*_G hot probe. A549 extracts are included in lanes 2 and 9 without the antibody and in lanes 3 and 10 with 0.3 ng of the MYB antibody. Daudi extracts are included in lanes 4 and 11 without the antibody and 5 and 12 with 0.3 ng of the MYB antibody. Jurkat extracts are included in lanes 6 and 13 without the antibody and in lanes 7 and 14 with 0.3 ng of the MYB antibody. As expected the specific protein–DNA complex is still observed in all cell lines tested. The addition of a MYB antibody (lanes 3, 5, 7, 10, 12, 14) results in the loss of the protein–DNA complexes.

Examining the ENCODE data for the region chr10:112,630,451–112,631,970 (GRCh37/hg19 assembly), the area including *rs6585018:G>A* has been reported to be rich in active regulatory elements, such as strong active enhancer regions, DNase I hypersensitivity sites and histone modifications as predicted by integrating chromatin immunoprecipitation sequencing (ChIP-seq) data [Dunham et al., 2012]. Transcription factor binding analysis revealed that one out of five *PDCD4* SNPs, *rs6585018:G>A*, had the potential to disrupt the binding of the transcription factor MYB (v-myb myeloblastosis viral oncogene homolog) (Supp. Table S4). An allele-specific band formation was found by EMSA using nuclear extracts from Jurkat and A549 cell lines. Competition assays in Jurkat (Fig. 3) and A459 cells (Supp. Fig. S3) revealed the formation of a protein–DNA complex specific for the A allele of *rs6585018:G>A* and identical results were obtained using a probe containing the MYB-consensus binding site (Supp. Fig. S4). This was further confirmed by supershift assays using anti-MYB in the reaction (Fig. 4) as well as assays in which a nonspecific antibody was included in the reaction (anti-SRY) (Supp. Fig. S5). Repetition of both the nuclear extractions and EMSA reactions gave identical results.

**Figure 5:**
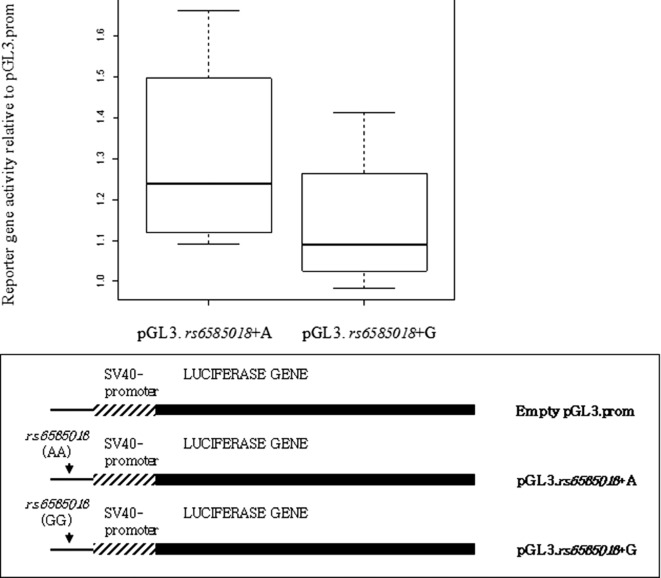
Reporter assay results comparing between Jurkat cell lines transfected with pGL3.*rs6585018*+A construct and pGL3.*rs6585018*+G construct. The means fold increase ±SD of five independent transient transfection experiments are shown. Paired *t*-test result was calculated comparing the mean luciferase activity of pGL3.*rs6585018*+A and pGL3.*rs6585018*+G in each experiment (*P < 0.02). Luciferase activity was normalized to *Renilla* and expressed relatively to empty expression vector. Mean luciferase activity relative to empty vector was 1.31 (SD 0.25) for pGL3.*rs6585018*+A and 1.14 (SD 0.19) for pGL3.*rs6585018*+G. The mean luminometer values of empty pGL3.promoter vector transfected alone into Jurkat cell line were 3030 light units. A representation of *rs6585018*+A and pGL3.*rs6585018*+G constructs is also provided.

To confirm the allele-specific effects of *rs6585018:G>A* polymorphism on promoter activity, two luciferase reporter constructs were generated including the MYB binding sequence 5′ of *PDCD4* and A/G at the *rs6585018:G>A* polymorphic site. After transient transfection into Jurkat cell lines, the relative luciferase activity of the pGL3.*rs6585018*+A transfected cells was found to be increased compared with pGL3.*rs6585018*+G transfected cells (*P* < 0.02) confirming the findings from the EMSA experiments (Fig. 5).

## Discussion

In this study, the findings of a GWAS in childhood asthma [Moffatt et al., 2007] were investigated in an independent group of severe asthmatic children and adults from the UK. Studying individuals with the severest form of asthma is of particular importance because of the high clinical costs associated with this patient group [Smith et al., 1997]. Our study identified a SNP, *rs6585018:G>A*, located within the predicted promoter region of the *PDCD4* gene on chromosome 10q24 to be significantly associated with childhood asthma (*P* = 0.001). The largest GWAS for asthma to date did not report the present association; however, severe asthma in adults and children was not assessed separately [Moffatt et al., 2010]. A recent GWAS examining severe asthma did not report any novel associations meeting genome-wide significance [Wan et al., 2012]. Using a *P* < 5 × 10^−5^ as a threshold for genome-wide significance, previously identified asthma-associated loci, *ORMDL3/GSDML* and *IL1RL1/IL18R1* were shown to be associated with asthma [Gudbjartsson et al., 2009; Moffatt et al., 2010]. The study however did not stratify for different age groups and the mean age of asthma-onset was 21 years.

Focusing our analysis in childhood asthma, we conducted fine mapping of the *PDCD4* region revealing two further SNP associations with severe asthma, *rs34104444:G>A* and *rs1322997:C>A* (*P* = 0.004 and *P* = 0.002, respectively). For all three SNPs, *rs6585018:G>A*, *rs34104444:G>A* and *rs1322997:C>A* belonging to the same haplotype block, minor allele frequencies G, A, and C respectively, were increased in asthmatics compared with the controls. Interestingly, the same SNPs showing association with severe asthma were also found to be associated with *PDCD4* transcript levels (http://www.sph.umich.edu/csg/liang/asthma), implying they have a functional role influencing the expression of the gene. The asthma-associated allele (G allele) in *rs6585018:G>A* was also significantly associated with higher IgE levels but not blood eosinophils counts and lung function in the combined children group. Severe asthmatic children have been generally associated more with atopic symptoms compared with severe asthmatic adults [Miranda et al., 2004; Wenzel, 2006].

We sought to explore these associations in an independent group of asthmatic children from Germany. In this population group, from all 14 tested SNPs in 10 genomic regions, *PDCD4* SNPs *rs1407696:T>G* and *rs11195360:T>C* were associated with total IgE levels. The same SNPs were only borderline-associated with IgE in the UK group of severe asthmatic children and controls, whereas *rs6585018:G>A* was not associated with neither doctor-diagnosed asthma nor total IgE in the replication group. These observations may reflect real genetic heterogeneity between the two populations or/and differences in phenotype definition. Indeed, asthma severity definition in the replication group did not follow the same guidelines as the severe asthmatic group from the UK. Nevertheless, *PDCD4* SNPs associations with the asthmatic status seen for the UK group and IgE levels seen for both children groups from the UK and Germany indicate that *PDCD4* is a locus of interest for the development of early onset severe asthma.

From bioinformatic analysis and EMSA results, SNP *rs6585018:G>A* was found to affect binding of the transcription factor MYB with the A allele having a higher affinity for MYB compared with the allele G which is also the asthma risk allele in the present study. The finding was confirmed by reporter assays indicating that MYB transcription factor exerts an allele-specific regulation of the expression of *PDCD4* gene. Of particular interest is the finding by GWAS that SNPs in the *MYB* gene (MIM #189990) confer susceptibility to eosinophil counts and asthma, providing further evidence that the MYB/PDCD4 mechanism may be of general importance to asthmatic inflammation [Gudbjartsson et al., 2009].

Myb transcription factor has been previously shown to induce *Pdcd4* expression in a chicken B cell line [Schlichter et al., 2001]. Using a B-lymphoid chicken cell line to disrupt *Myb* gene by homologous recombination resulting in Myb knock-out, *Pdcd4* expression was diminished, providing additional evidence of the role of the human *MYB* in the regulation of *PDCD4* expression [Appl and Klempnauer, 2002]. The presence of MYB regulatory elements in *PDCD4* promoter suggests that the human gene could be also under MYB-transcriptional regulation. A recent study on the promoter of *PDCD4* revealed strong regulatory elements adjacent to the MYB binding site including *rs6585018:G>A* [Leupold et al., 2011].

PDCD4 is expressed in proliferating cells and protein levels are modulated by IL-12 and IL-2 [Azzoni et al., 1998]. The protein interacts with translation factor EIF4A through two MA-3 domains inhibiting the initiation of translation [Yang et al., 2004; Yang et al., 2003]. Interestingly, target mRNAs of PDCD4 include IL-4 and IL-10 [Hilliard et al., 2006]. PDCD4 acts as a transcription regulator of mRNA molecules such as the urokinase receptor u-PAR [Leupold et al., 2007], which has been found to influence the eosinophilic adhesion in asthmatics [Brooks et al., 2006]. Recent studies have shown that PDCD4 takes part in the negative regulation of TLR4 signaling, a prominent pathway in allergic asthma [Sheedy et al., 2010]. Down-regulation of PDCD4 leads to the establishment of an inflammatory environment (IL-10, IL-6, TNF-α) [Yasuda et al., 2010], suggesting a role in fine-tuning inflammatory events. In the study by Sheedy et al., downregulation of PDCD4 expression was mediated via miR-21, a molecule found to be up-regulated in airway inflammation [Lu et al., 2009; Moschos et al., 2007]. Negative regulation of PDCD4 by miR-21 could also impact other pathophysiological characteristics of asthma, like smooth muscle contractibility [Davis et al., 2008]. PDCD4 has been widely studied as anti-cancer therapeutic target [Lankat-Buttgereit and Goke, 2009]; its role however as a translation inhibitor in inflammation, cell invasion and smooth muscle contraction could be indicative of its implication in the expression of the asthmatic phenotype.

The results from this study have shown that the putative promoter-located *PDCD4* SNP *rs6585018:G>A* is associated with severe asthma in children and that it could influence the transcription of the *PDCD4* gene in an allele-dependent manner. Allele G associated with severe asthma and higher total IgE levels leads to less MYB binding and therefore lower PDCD4 expression as it is evident by the functional analyses. Other studies using various cell types have shown that the regulation of *PDCD4* expression can occur at many levels including transcriptional and translational and that these mechanisms could be essential for a coordinated and controlled regulation of the cellular protein levels [Lankat-Buttgereit and Goke, 2009]. Importantly, the present study highlights the regulatory role of MYB in *PDCD4* transcription, which needs to be considered in future functional investigations of this molecule.

Our study has a number of limitations. First, the sample population of severe asthmatic children is small (*N* = 116) explained by the low frequency of this phenotype. However, our strategy enabled us to investigate the extreme differentiated asthmatic phenotype and control for the homogeneity of the selected cases, since they were recruited from the same pediatric clinic. Our study focused on *PDCD4* SNP rs6585018*:G>A* as the one significantly associated with severe asthma and also predicted to have a functional role; however, due to the small size of our severe asthmatic children group, we cannot fully disregard the rest of the GWAS hits as nonassociated with asthma. Second, the frequency of the risk allele G of SNP *rs6585018: G>A* is low in the general population (CEU-HapMap; MAF: 0.06), limiting the wider significance of the finding. Nevertheless, rare variants have been proven valuable in highlighting novel mechanisms underlying diseases [Weidinger et al., 2008]. The association was not reported in the largest asthma genetic study at present including twenty thousands of cases and controls, possibly because it included only a small number of severe asthmatic children [Moffatt et al., 2010]. An important following step would be to further examine these findings in additional panels of severe asthmatics, with subphenotypes of lung function measurements, blood eosinophils and most importantly atopic status to confirm the role of *PDCD4* SNPs associations and clarify the functional relevance of the molecule.

In conclusion, this study combining a genetic analysis in well-defined population of severe asthmatics and controls, incorporating functional approaches reports that *PDCD4* locus is associated with severe asthma and IgE levels, whereas SNP *rs6585018:G>A* exerts a regulatory effect on *PDCD4* expression. We therefore propose that the PDCD4 protein and MYB-dependent regulation would be worthwhile for further investigation of its role in asthma-related mechanisms.

## Supporting Information

Disclaimer: Supplementary materials have been peer-reviewed but not copyedited.
