# Supplementary material for: A Polymorphism Affecting MYB Binding within the Promoter of the PDCD4 Gene is Associated with Severe Asthma in Children
Source: Hum Mutat. 2013 May 20;34(8):1131–9. doi: 10.1002/humu.22340 (PMC4296222; doi:10.1002/humu.22340)
Supplement: Supplementary file 1 — supplementary material [file humu0034-1131-sd1.pdf]

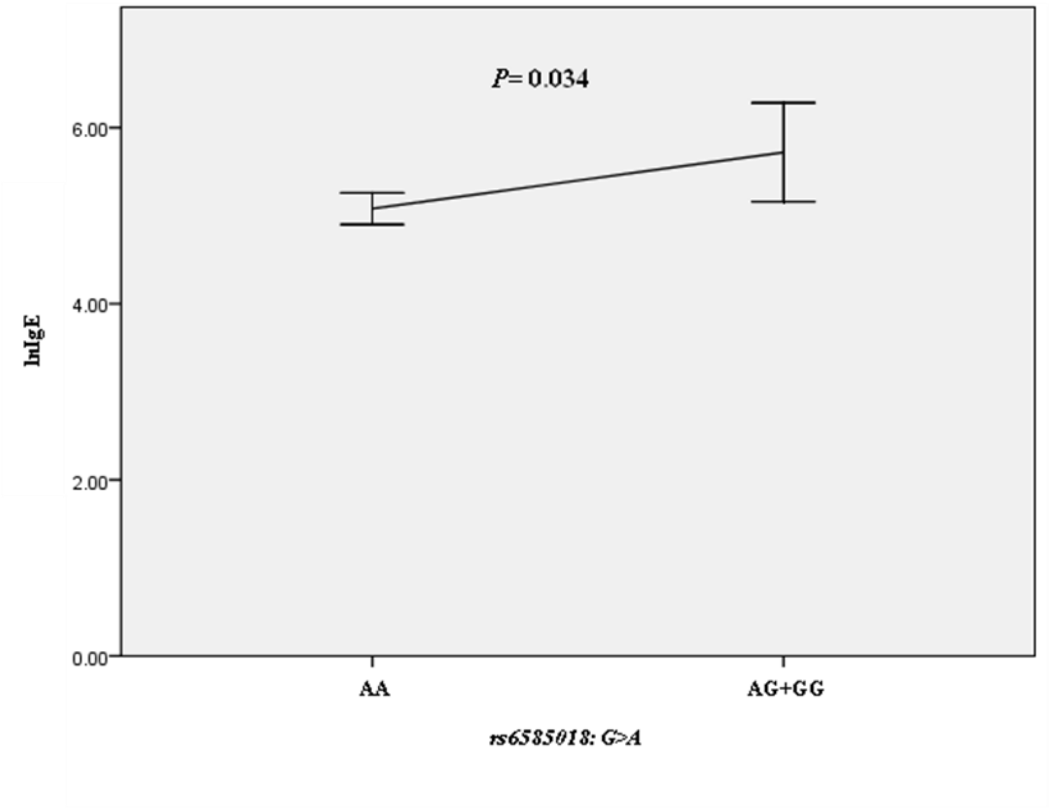

Supp. Figure S1. Means plot for total IgE in all children combined group (N=556) by *rs6585018: G>A*. IgE was log-transformed as lnIgE. Subjects were analysed by dividing them in those carrying the G risk allele (AG+GG group) and those not carrying the G risk allele (AA). Means of lnIgE for (AA): 5.1 (95% CI: 4.9-5.2) and means of lnIgE for (AG+GG): 5.7 (95% CI: 5.2-6.2), *P* value= 0.034).

a.

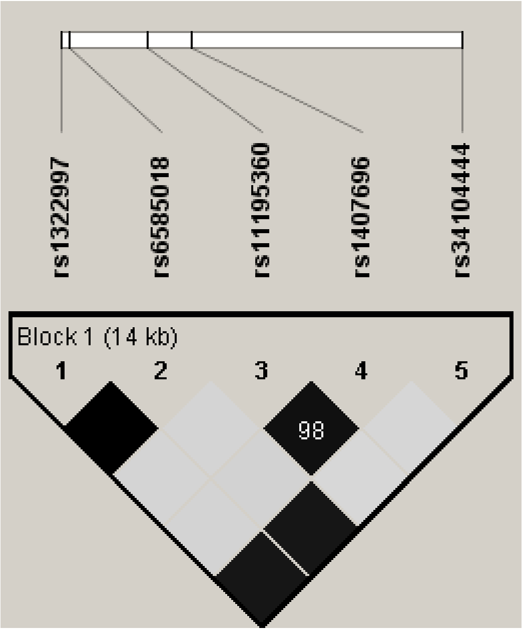

b.

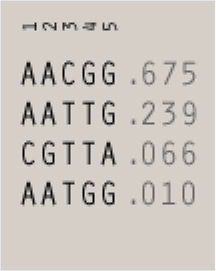

Supp. Figure S2. a. The haplotype structure of the 5 *PDCD4* SNPs (*rs1322997:C>A*, *rs6585018:G>A*, *rs11195360:T>C*, *rs1407696:T>G* and *rs34104444:G>A*) as constructed in Haploview. The r^2 values for linkage disequilibrium are shown in a grey scale plot (white: r^2=0, black: r^2=1), **b.** the combination and frequencies of the haplotypes of the 5 SNPs.

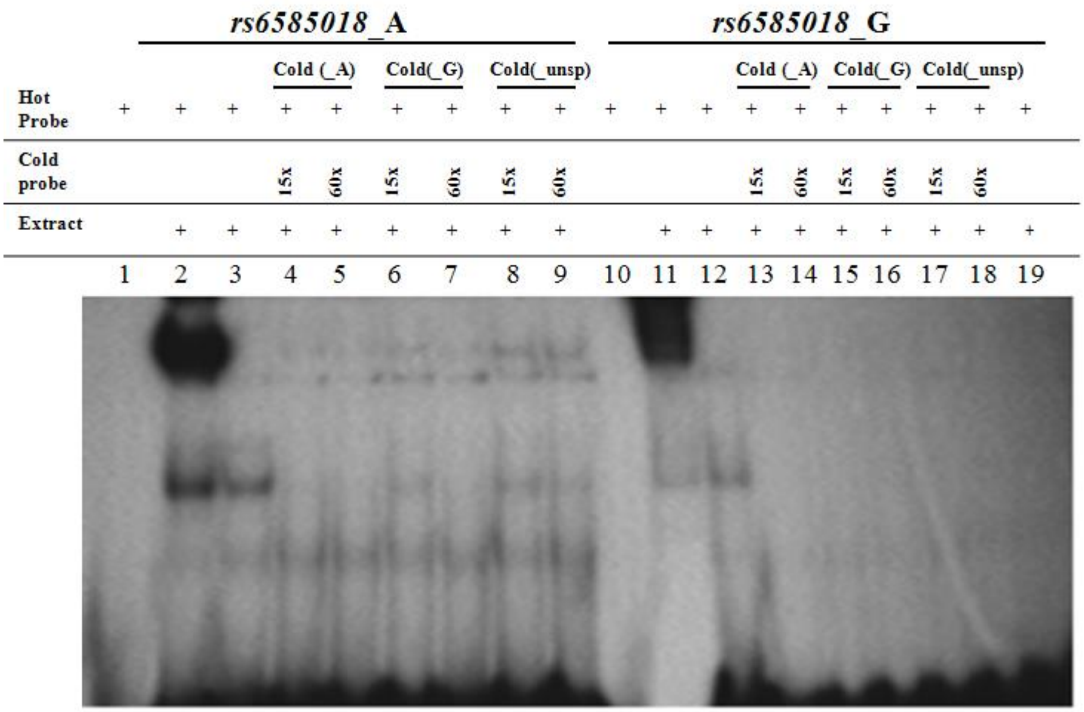

Supp. Figure S3. Competition assay for *PDCD4* SNP *rs6585018:G>A* using 10μg of A549 nuclear extract per reaction. Lanes 1-2 include only the *rs6585018*_A hot probe (with and without non-specific co-polymer, NC). Lanes 10-11 include only the *rs6585018*_G hot probe (with and without non-specific co-polymer, NC). Lanes 2-9 include the *rs6585018*_A hot probe and lanes 11-18 the *rs6585018*_G hot probe with protein extract. Lanes 3 and 12 include reactions with the hot probe and the extract only. Unlabeled probe in excess was added in the competition assays (lanes 4-9 and 13-18) as follows: lanes 4 and 13-15X and lanes 5 and 14-60X of unlabelled *rs6585018*_A, lanes 6 and 15-15X and lanes 7 and 16-60X of unlabelled *rs6585018*_G and lanes 8 and 17-15X and lanes 9 and 18-60X of unlabelled *PDCD4* _unspecific probe. Lane 19 contains a reaction with a hot unspecific probe predicted not to bind to any proteins. As expected no protein-DNA complex was formed in this lane.

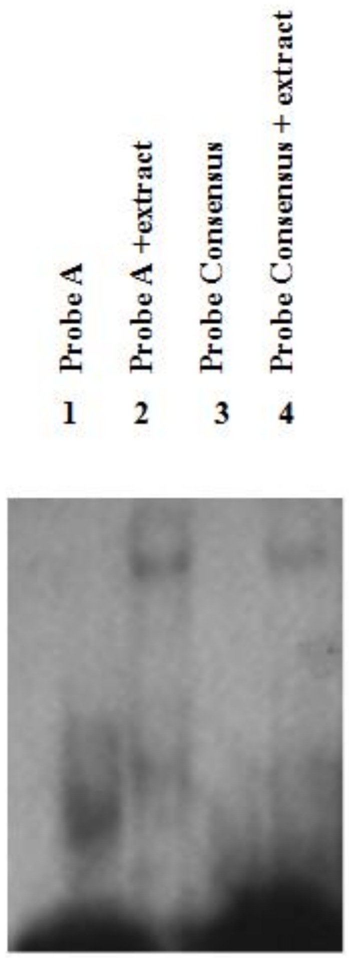

Supp. Figure S4. EMSA reactions including a MYB-consensus probe. Lane 1 and 3 contain reactions with *rs6585018*_A and MYB-consensus probe respectively and lanes 2 and 4, 10 µg of Jurkat nuclear extract is added in the reaction.

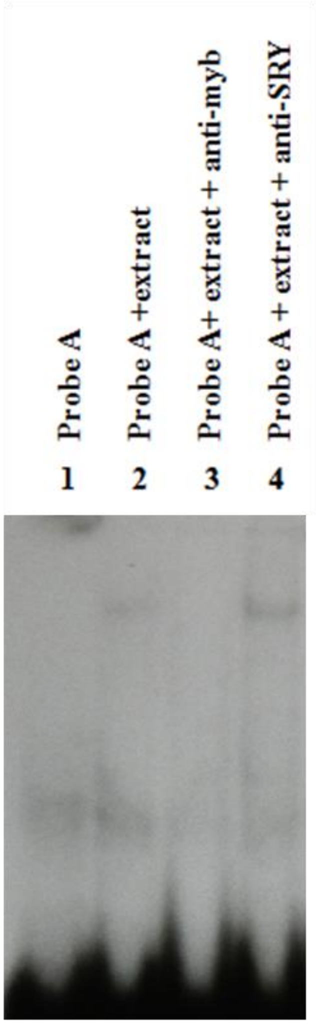

Supp. Figure S5. Supershift assays with *rs6585018*_A including a specific (anti-MYB) and unspecific antibody (anti-SRY). Lane 1 includes the probe, lane 2 includes 10 μg of Jurkat nuclear extract and in lanes 3 and 4 anti-MYB and anti-SRY is added to the reaction respectively.

Supp. Table S1. Genotyping results for the 5 total *PDCD4* SNPs from the fine mapping study part

**SNP**	**Location**	**Alleles (Minor/Major)**	**Call rate**	**HWE χ^2 test *P* value**
rs1322997:C>A	5’ near gene	C/A	99.1%	*P*=0.58
rs11195360:T>C	Intron 1	C/T	98.1%	*P*=0.95
rs1407696:T>G	Intron 2	A/C	97.8%	*P*=0.73
rs34104444:G>A	Exon 5 / Synonymous	A/G	98.6%	*P*=0.97
rs6585018:G>A	5’ near gene	G/A	99.2%	*P*=0.88

HWE: Hardy-Weinberg Equilibrium

Supp. Table S2. Association test results for SNP *rs6585018:G>A* for all children group

rs6585018: G>A	**G allele frequency**	**OR (95% CI)**	**Fishers exact test *P* value**
**All asthmatic children combined : Healthy children 411:145**	0.08 : 0.04	1.99 (1.07-3.81)	*P*=0.02

OR: Odds Ratio; CI: Confidence Intervals

Supp. Table S3. Association results for IgE levels and all SNPs tested in the study

SNP	Gene	Severe Asthma 1 + Controls		Severe Asthma 2 + Controls	
		N	*P* value	N	*P* value
rs6656822:T>C	*SLC19A2*	789	0.0541	**689**	**0.0444**
rs1401107:C>T	Intergenic	789	0.1214	689	0.5175
rs1356847:T>C	Intergenic	789	0.6095	689	0.6126
rs12715305:C>G	*DLEC1*	789	0.6118	689	0.6522
rs11097415:G>A	*SHROOM3*	789	0.3884	689	0.1754
rs10270097:C>A	*DGKI*	789	0.2732	689	0.6589
rs1322997:C>A	*PDCD4*	789	0.6337	689	0.4171
rs6585018:G>A	*PDCD4*	789	0.6480	689	0.4313
rs1407696:T>G	***PDCD4***	**789**	**0.0111**	**689**	**0.0063**
rs11195360:T>C	***PDCD4***	**789**	**0.0212**	**689**	**0.0145**
rs34104444:G>A	*PDCD4*	789	0.5963	689	0.3876
rs481297:T>C	*ST8SIA5*	789	0.6751	689	0.6707
rs248944:C>G	*ZNF506*	789	0.4560	689	0.7290
rs2243603:G>C	*SIRPB1*	789	0.9688	689	0.6069

Results were obtained for combined cases and controls. The significant results are shown in bold. (Severe Asthma 1=asthmatic children with more than 1 hospital admissions during the last 12 months; Severe Asthma 2= asthmatic children with at least 4 hospital admissions during the last 12 months; N=number; *DGKI: Diacylglycerol Kinase Iota*; *ST8SIA5: ST8 alpha-N-acetyl-neuraminide alpha-2,8-sialyltransferase 5*; *SIRPB1: Signal-Regulatory Protein Beta 1*; *DLEC1: Deleted in Lung and Esophageal Cancer 1*; *SHROOM3: Shroom family member 3*; *SLC19A2: Solute Carrier family 19 (thiamine transporter), member 2*; *PDCD4: Programmed Death Cell Domain 4*; *ZNF506: Zinc Finger protein 506*).

Supp. Table S4. Results from the analysis of the *rs6885018:G>A* SNP sequence for transcription factor binding sites

**Allele**	**Program**	**TF**	**Strand**	**Position**	**Sequence**	**Score**
rs6585018_A	TFSearch	HSF	+	8-12	AGAAAG	100.0
		v-myb	+	12-20	AGCAACGGC	92.0
		c-myb	-	10-27	AAAGCAACG GC CAGAGGG	87.6
		ADR	+	25-29	GGGAG	86.2
	MatInspector	Cellular and viral myb-like transcriptional regulators	+	13-25	G**CAA**CGGCC AGAG	0.985
rs6585018_G	TFSearch	HSF	+	8-12	AGAAAG	100.0
		ADR	+	25-29	GGGAG	86.2
	MatInspector	-	-	-	-	-

The SNP position is indicated in red. The bases in bold on the MatInspector results are the core binding sites for the transcription factors. As for the transcription factors that bind to the sequence, only myb appears to exhibit alternate binding between the different alleles of *rs6581018:G>A* (ADR: Adrenergic Receptor; HSF: Heat Shock Factor; TF: Transcription Factor).
